# A qualitative investigation of exercising with MS and the impact on the spousal relationship

**DOI:** 10.1186/s11556-015-0148-5

**Published:** 2015-10-07

**Authors:** Sean Horton, Dany J. MacDonald, Karl Erickson, Rylee A. Dionigi

**Affiliations:** Department of Kinesiology, University of Windsor, 401 Sunset Ave., Windsor, N9B 3P4 Canada; Department of Applied Human Sciences, University of Prince Edward Island, Charlottetown, Canada; Michigan State University, 308 West Circle Dr., East Lansing, MI 48824 USA; School of Human Movement Studies, Faculty of Science, Charles Sturt University, Panorama Avenue, Bathurst, NSW, 2795 Australia

## Abstract

**Background:**

Multiple Sclerosis is an autoimmune disease that affects more than 2.3 million people around the world. Symptoms are numerous and varied, often having a profound effect on activities of daily living. While for many years individuals with MS were told to avoid exercise for fear of worsening their symptoms, recent research has emphasized the multi-faceted benefits associated with regular physical activity. Given the strain that MS can put on family and interpersonal relationships, the intention of this study was to investigate the exercise experiences of individuals with MS and the extent to which these experiences affect, or are affected by, their spousal relationship.

**Methods:**

In-depth qualitative interviews were conducted with 10 individuals, five with MS, along with each of their spouses, in order to gain a comprehensive understanding of living and exercising with the disease. An inductive approach was used to analyze the interview data.

**Results:**

The results displayed the important physical, psychological, and social benefits of involvement in an exercise program. Spouses help to counteract barriers and facilitate exercise, and are well aware of the integral role they play in their partner’s health and well-being. Spouses also valued the increased independence they gained, in the form of reduced care-giving responsibilities and enhanced social opportunities, as a result of the improved physical function of their partner. These findings contrast the severe strain on spousal relationships that is often reported in studies on people living with MS.

**Conclusions:**

Rather than an inexorable downward decline in physical ability that is common with MS, participants spoke of a positive reversal in physical function, which has had far-reaching implications for multiple aspects of their lives, including their psychological outlook, their sense of independence, overcoming isolation, and their relationship with their spouse, all of which are identified in the literature as notable aspects of life affected by the disease.

## Introduction

Multiple Sclerosis (MS) is an autoimmune disorder that affects the central nervous system. The disease results in the destruction of myelin, which gradually but inexorably inhibits motor coordination and control. Additional symptoms include increased fatigue, lack of coordination, impaired sensation, sensitivity to heat, vision problems, and balance-related issues [1]. MS is a global disease, with more than 2.3 million individuals affected [2]. While there is no cure at present for MS, and there are implications with respect to life-span, people with the disease can expect to live a long time after being diagnosed. Many of those years will encompass financial strain. Of the 700,000 individuals in Europe with MS, 50 % become unemployed within three years of their diagnosis [3]. Canada has the highest rate of MS in the world, with an accompanying unemployment rate of 80 % [4].

The onset of MS is predominantly between the ages of 15 to 40, although due to difficulties in detection, diagnosis often occurs later in life [5]. Those individuals who contract MS are at risk of diseases associated with sedentary living, due to the fact that the symptoms of MS, primarily fatigue and heat sensitivity, often make exercise and physical activity uncomfortable [6]. In fact, for many years, people with MS were advised to avoid exercise due to those associated symptoms [7, 8]. Recent estimates suggest that 78 % of individuals with MS get no exercise at all, which compares unfavourably to the 38 % of the general population considered to be inactive [9]. Thus, chronic conditions associated with a physically inactive lifestyle (obesity, cardiovascular disease, type 2 diabetes) are often correlates of having MS [10]. Due to these risks associated with inactivity, people with MS are more prone than the general population to experience health complications that results from a sedentary lifestyle [11]. More recent research has challenged early recommendations that people with MS avoid physical activity [12, 13]. Exercise is now considered to be an important part of MS symptom management. Guidelines published in 2013 provide exercise prescription specific to individuals with MS, albeit at levels lower than recommended for the general population [9, 13].

Given the relationship between MS, aging, exercise, and the potential for engagement in physical activity to improve fitness levels and quality-of-life measures [10, 13] it is important to understand the complexity inherent in the decisions people with MS make with respect to their involvement in any kind of exercise program. Significant barriers beyond MS symptom management exist for those who do partake, or want to participate, in physical activity. Borkoles and colleagues noted that there is little understanding within the health and social service community on how to adequately support those with MS who wish to be physically active in spite of their symptoms [14]. The authors utilized qualitative interviews to examine the lived experiences of seven participants diagnosed with MS as it related to their involvement with exercise. One noteworthy theme to emerge from their interviews was participants’ frequent dependability on others to overcome the challenges they faced. Subsequent focus groups conducted by Learmonth and colleagues emphasized the important social component associated with a group exercise program, and the authors reiterated that both the symptoms of the disease and the lack of support services made exercise problematic [15].

There is a growing literature on the multi-faceted role of the spouse or caregiver to individuals with MS. While couples dealing with MS are aware of pending health changes, planning for these changes is frequently avoided [16]. The reasons for this are numerous and multi-faceted. Rates of depression are high for those people living with MS [17] and depression negatively affects communication between partners [18]. Courts and colleagues noted that while people with MS are traditionally prescribed medication for their depression, many expressed a desire for support rather than prescription drugs [19]. Not surprisingly, MS affects the quality of life of both individuals in a partnership [17] and the corresponding negative effects have been referred to as the ‘third person’ in the marriage [19].

There has, however, been very little investigation into the role of the spouse as it relates to exercise involvement. Considering the extent to which exercise can improve health outcomes and potentially alleviate symptoms of depression [20] and the acknowledged role of the spouse as advocate and protector of their partner [19] understanding the potential facilitative capabilities (or hindrance) of spouses as they relate to exercise involvement is worthy of enquiry. To this end, the purpose of this study was to investigate the exercise experiences of individuals with MS and the extent to which these experiences affect, or are affected by, their spousal relationship.

## Methods

Owing to the dearth of research on this topic, this study takes an exploratory, qualitative approach, with no explicit theory or hypothesis set out in advance, but becomes progressively sharpened as the study proceeds [21]. In-depth qualitative interviewing is characterised by receptive, careful listening, open-ended questions, and additional probing on the part of the interviewer [22]. Interviews with people with MS and their spouses (who did not have MS) were conducted in a semi-structured format [23], which provides the interviewer the flexibility to probe participants’ answers for more detail. An interview guide provided the basic topics, which included questions about their current exercise regime, overcoming barriers to exercise, and the extent to which exercise has had an impact on their quality of life. These key topics were discussed with all participants, although the semi-structured interview permitted the exploration of new issues significant to the participant that emerged during individual interviews [23].

### Participant recruitment procedure

The lead author’s former involvement in an exercise program provided the initial contacts. The program includes group members both with and without MS. This group has scheduled workouts twice per week, and while they meet as a collective, each member has his or her own individualized exercise program that they follow. Workouts generally last for one hour. The three members in the group who have MS were approached and agreed to take part in the study. In addition, we asked each of their respective spouses to participate in an interview, as this would help to provide a more holistic representation of living with the disease and gain their perspective on the role they might play in their spouse’s exercise involvement.

In order to gain a more complete picture of exercising with MS, two participants who exercise on their own (i.e., not as part of the abovementioned program) were also recruited through our connections within the MS community. Each agreed to discuss their exercise experiences. One participant plays recreational ice hockey and attends a gym once or twice a week for workout sessions. The second goes intermittently to a gym to work out on his own. Once again, we invited their respective spouses to participate in an interview.

### Interview format

All of the interviews took place in a private room with one interviewer. While the exact sequence and wording of questions varied, questions to the participants with MS were aimed at understanding their current exercise regime and the extent to which their involvement in exercise affected their quality of life. Specifically, we were interested in how exercise affected both physical measures (i.e., activities of daily living) and participants’ psychological well-being. We were also interested in the challenges that people with MS encounter with respect to exercise, and how they attempted to meet those challenges.

The interviews with the spouses were done separately. Each spouse was asked a similar set of questions revolving around the aforementioned topics (regarding their partner’s exercise involvement) in an attempt to obtain a more complete picture of living with MS. In particular, we wanted to gain insight into the spouses’ perspective on how they believed exercise affected their partner’s physical and mental well-being, as well as the challenges that their partner faces. Interviews with each of the participants lasted from 40 to 75 min, with most interviews taking approximately 60 min.

### Participants

The university hosting the exercise program provided ethical clearance for the study through their research ethics board. Participants were assured that every possible strategy would be utilized to protect their anonymity, and they all provided informed consent. We have given each participant a pseudonym to help protect their identity. In total we interviewed five participants with MS (four male, one female), all of whom ranged between four and six on the Kurtzke Expanded Disability Status Scale (EDSS) [24]. Four on the EDSS indicates significant disability but generally self-sufficient and able to walk 500 m without aid or rest. A score of six indicates greater mobility challenges; walking 100 m with our without rest generally requires the use of a walking aid (i.e., cane or crutch) [24]. The mean age was 57.4 (SD = 11.6, age range 45–70) and the average age that participants were diagnosed with MS was 44 years of age (SD = 12). In addition, we interviewed each of the spouses (one male, four female, mean age = 56.8, SD = 11.7, age range 44–69), who did not have MS, for a total of 10 interviews. All participants identified as Caucasian, and while the diagnosis of MS had, in some cases, affected their employment status, SES levels ranged from moderate to above average based on their stated professions.

### Analysis

Each of the 10 interviews were transcribed verbatim and corrected against the audiotapes by the lead author. Based on the questions that were asked and numerous readings of the interviews, broad themes for the responses were established that provided a preliminary framework. In accordance with the hierarchical content analysis outlined by Côté and colleagues [25, 26], an inductive approach was then used in which comments and quotes from the interviews were coded as “meaning units.” Subsequently, common features between meaning units were identified. This procedure involved comparing and organizing meaning units into distinct groups [25, 27]. For example, a statement such as ‘my energy level is sometimes just so low’ was coded as an individual meaning unit. Similar statements that related to issues concerning energy level and fatigue were grouped together, eventually constituting the category *managing energy levels*. This category was later encompassed within the major theme, ‘Negotiating if exercise is worth it’.

Separate documents were created for each of these groups, or themes. These themes remained flexible during the investigation and were continually refined and debated amongst the co-authors until a classification system was agreed upon that best represented the qualitative material, a process referred to as the constant comparative method [28]. This process was initially carried out within each interview (i.e., intratextually) and then across interviews (i.e., intertextually) [29]. The outcome was three broad themes with several categories (or subthemes) within them. This approach to analysis ensured that the findings remained grounded in the data and allowed common themes to emerge across the entire data set representing the exercise experiences of individuals with MS and the extent to which these experiences affect, or are affected by, their spousal relationship [28]. In particular, this analytical strategy allowed us to understand the data in terms of exercise and its effects on physical and mental well-being, as well as how people with MS (and their partners) manage challenges in relation to exercise.

### Trustworthiness

Expert-checking was incorporated by having experienced qualitative researchers involved in the development of the interview protocol and by consulting with them throughout the coding process [30]. Ongoing discussions between the authors enabled critical reflection on the analysed data until codes and themes were agreed upon. In addition, member-checking was employed by contacting the participants after the interview to invite them to add to or amend any of their responses.

## Results

Overall, three interrelated major themes emerged from the analysis: *Maintaining independence, Overcoming isolation,* and *Negotiating if exercise is worth it.* Within two of these major themes a number of categories emerged and each of these will be discussed in turn. For clarity purposes, any quotes for which it is unclear whether the speaker is the individual with MS or the spouse, we have indicated spouse next to the name in parentheses.

## Maintaining independence

The desire to maintain a sense of independence was one of the most powerful and consistent themes that came out of our participant interviews. Within this major theme, three categories emerged during analysis: the *effect of MS on the spousal relationship, exercise improving physical functioning,* and *exercise affecting psychological outlook.*

### Effect of MS on the spousal relationship

The onset of MS has a multi-faceted impact on families, affecting their physical, psychological and emotional lives and often taking a major toll on relationships. Louise (spouse) spoke of the anger that often accompanies a diagnosis of MS:

One of the things that I realized about people with MS and their families, especially partners, is that there is an anger. One man with MS we know, younger, like mid-thirties, early forties and he’s now on disability. The anger is increasing in that family and while his wife is dealing with the anger, he’s retreating. He’s doing what I saw (my partner) doing a few years ago.

Nora (spouse) explained the frustration she has experienced, and how, in addition to the increased workload around the home, it has curtailed some of her freedom and independence:

It’s affected me a lot because (Alex) is limited in what he can do, his stamina, so I have to do everything. Even as simple as now I have to put out the garbage too, or shovelling all the snow, all the lawn maintenance. If I just want to go for a run, I have to either ask someone to baby-sit, depending on how he’s feeling. I can’t be spontaneous, that’s the part that I find the worst. If people phone me and say, ‘Hey, do you want to go out?’ and I’ll be like… ‘I have to see if I can get a babysitter’. So, yeah, that’s a huge impact on me.

John (spouse) acknowledged how close he and his wife were to a complete breakdown of their relationship, due to her deteriorating condition and the mental toll it was taking on both of them.

We were both to the point where we were almost ready to divorce or separate because of her disease taking her down. I had to do everything, all the housework, and laundry and sometimes helping her get dressed and for a while, after she broke her ankle, we were really, really in a bad place for a while.

The subsequent involvement of this couple in the exercise program has paid substantial dividends, as John believes that Karen’s increased physical strength has provided her a degree of independence. In the following quote he describes the feelings of guilt he suffered any time he left Karen alone, but how this has eased as her physical functioning improved.

I couldn’t leave her and I had no one to come and stay with her. I felt guilty going and leaving her, getting out and doing something and not being able to take her. And I still feel that way. I go sometimes with my buddies for a fishing weekend and I worry about her while I’m gone. Of course, I have a phone so we can get in touch but I just feel guilty when I have a little bit of fun and I don’t take her. I don’t think I’ll ever get rid of that. But it’s not too bad now that I know she can get around the house with a walker and cook her own meals and take care of herself.

All of the participants with MS, irrespective of their current exercise regime, saw the value of partaking in physical activity and this finding is exemplified in the category *exercise improving physical functioning*. In addition, each spouse fully endorsed their partner exercising.

### Exercise improving physical functioning

The improvements the participants experienced, and attributed specifically to their exercise regime, were often dramatic. Ben commented on how close he had been to full-time confinement to a wheelchair before he started exercising regularly.

I just got progressively better and better and better because of doing the exercises. Before that I was using a scooter and I was basically in and out of a wheelchair and going downhill quite rapidly.

Karen noted similar dramatic improvement in her physical functioning.

When I started (in the exercise program), I could hardly walk from my bedroom to the bathroom and stay up on my feet. I had to struggle to stay on my feet to get from the bed into the bathroom, struggle getting off the toilet, struggle to stand and wash my face and brush my teeth, and now I can do all those things. I’m starting to do some cleaning and tidying. It’s just amazing, the difference in my body.

This change in functioning was reiterated by her spouse, who noticed the distinct transformation after his wife started exercising on a regular basis.

I couldn’t believe when she started exercising how her strength came back and her balance. She was actually able to do stuff around the house like she used to, like the laundry and help with dishes and stuff like that. It was just amazing (John).

Without exception, all of the participants commented on the positive impact exercise had on their physical functioning, and this finding was affirmed by their respective spouses.

### Exercise affecting psychological outlook

Just as notable as physical benefits was the extent to which exercise positively affected participants’ state of mind, and importantly, their perceived quality of life. Small improvements in physical strength translated into meaningful improvements in day-to-day living. Spouses reiterated the positive effect that exercise had not just on their partner’s physical health, but also on their attitude.

He comes home positive. He feels better about himself, he feels that he’s accomplished something. He notices that he can do things better, just simple tasks that he would do every day that he feels more confident about. Even our interpersonal relationship, he’s more positive, he’s more engaged. He comes home happier (Louise)

Karen explained:

This program, it’s not just the exercise, even though it’s a big thing, … I felt I was a prisoner in my own home the last year, before I started this program. I had anger issues, I had frustration issues. I would stand at the window and look out and think ‘I want to just walk down my driveway and get the mail’. You can’t even step outside without help. We have a beautiful great big double-deck on one side of our house and a beautiful deck out the front, and I couldn’t go out there and sit on a chair without help. But last summer, I did. I was able to. I felt a little bit freer. I’m starting to get a little bit of independence back…. There are two friends of ours that we’re very close to, but I hadn’t been to their places in a year because both of them have a lot of stairs in the houses, but now I’m back to visiting them.

This quote exemplifies the intense frustration so common for individuals with MS, but of significance to our study is the extent to which this individual’s involvement in exercise (like others in the sample) has improved her physical function, re-established a measure of independence, and changed her outlook. It also illustrates the social implications of MS, and the extent to which faltering physical function can limit social opportunities. This outcome relates to our next major theme; overcoming isolation.

## Overcoming isolation

The participants with MS, particularly those who were involved in the structured group-based exercise program, emphasized the social aspect and how that was beneficial above and beyond the exercise itself.

Belonging is an important thing. You belong because you’re doing the exercises and you’re sharing it with everyone. People ask “How you doing today?” “Oh, I did this…” “Oh, yeah, did you?” You’re sharing all that. It’s a bit of a bond. (Rob)

Ben also mentioned this sense of belonging, and that the social component of group exercise was a crucial part of the program for him:

I talked to a lady (with MS) and she tries to do some exercises on her own. But there’s a social network and if you can do it as a group, it’s just so much better for you. You’re in a relationship with people, you can see them doing it and you want to work harder to do it yourself. I don’t know how people do it all alone in their room.

Spouses were also mindful of the social aspect of exercise, and the potential of keeping their partners engaged in the outside world. All of the spouses were particularly attuned to the notion of isolation, and the tendency for their partners to withdraw from social activities. Betty (spouse) explained:

I realized that he was staying at home, he wasn’t doing anything, he was isolating himself and I think that’s both emotional and physical. He didn’t feel well with the fatigue, it hurt to move, he didn’t want to do anything, he didn’t want to go anywhere. So there was both the emotional and physical aspects of everything that was happening to him.

The exercise program provided a purpose, a reason to get out of the house. As Louise (spouse) noted: “I think people with anything wrong with them, if they end up sitting home and don’t get out, don’t talk to different people, don’t do different things, they waste away”. For some of the spouses, the social aspect was just as important as the exercise itself, and a crucial part of dealing with the disease:

Part of that is the social part, just the psychological part of going and accomplishing something and feeling good about it. The exercises have certainly strengthened (Rob) but one of the other aspects of going to that program is that it gets him out of bed. He has a reason to go somewhere. Being with people has been really helpful. MS is an isolating disease. You can very quickly feel so awful that you don’t want to do anything, but it’s that stimulation that gets you up and going and you get to talk to somebody else - that’s beneficial too (Betty).

Importantly, spouses acknowledged that the isolation affected not just their partner with MS, but them as well. Often this was related to the extra duties they took on after their partner’s diagnosis, which ultimately affected their social lives. John (spouse) noted the extent to which his social life suffered, to the point that he considered himself imprisoned in their home.

I wish they could get somebody they could have come in and give the care-giver a day off … just let them get away and do something and know that whoever’s there is going to be able to take care of things for a while. I felt like I was in prison almost. I wasn’t seeing my friends, talking to them, I wasn’t getting out. It would be nice to have somebody come in and spell me off for a few hours, so I could just go out and get away for a while, you know, just go to town or whatever and walk around the mall.

Many of the participants, the spouses in particular, felt that support from the MS community could play an important role in helping families adjust and adapt, by alleviating some of the things that can lead to isolation. Nora (spouse) suggested:

Maybe MS societies have to provide more client services to their patients, so that they can have someone come in and cut the grass or have someone come in and shovel the snow or have someone provide babysitting service for the kids, like twice a week.

At the same time, the spouses were cognizant of the important role they themselves could play in terms of facilitating their spouses’ involvement in exercise and helping to overcome isolation. Louise took the initiative by signing them both up for the exercise program, and acknowledged it was probably not something Ben (with MS) would have done on his own. Louise noted:

I think it’s easier for (Ben) to go because I’m going with him. There’s another fellow who comes who also has MS, but his wife doesn’t come and so he doesn’t come nearly as much. And, I’m not sure that (Ben) would get up at 6:30 on his own twice a week if I weren’t going with him. I think that support of having somebody else – friend, partner, whoever - doing it with you is really important.

Similarly, Adriana (spouse) considered it part of her role to get her husband to the gym.

I try really hard to make sure that (Jeff) goes to the gym when I go to the gym. If I find he’s not gone for a couple of days, I say “come with me” and he always feels better once he’s done it. I really try to focus on the fact that we’re going to exercise and eat well and sleep well, which is the job of any partner I think.

Clearly, exercise was considered by all of our participants as an important investment in health and well-being, but also as a way of maintaining connections with the world outside of their own home. The barriers to exercise can be significant for individuals with MS, however, and are easily underestimated by those unfamiliar with the disease. The mobility issues that people with MS face often contribute to their withdrawal from activities, thus making them more prone to feeling isolated. Transportation to and from an event or activity is an important consideration and often inhibits participation, particularly if it requires a long walk to the exercise facility. As Alex noted; “Parking’s a huge problem for people coming say in handicap vans, or coming so they can park close to the facility”. This finding leads us to our final major theme, in which participants grapple with the pros and cons of exercise.

## Negotiating if exercise is worth it

Resources that are available to people with MS are not always convenient, at least in terms of time and efficiency. While Jeff remarked that there are services in place to help individuals with MS, an hour of exercise can turn into the better part of the day when transportation issues are factored in.

If you want to go by access bus to get to these places, you have to book the access bus two weeks in advance and then there’s no guarantee you get picked up at the right time to come home again. So now the fitness thing could end up being five hours, you know, so there’s a whole bunch of things that factor into any kind of fitness program for people with disabilities.

This is an example of the struggle our participants faced, and the cost-benefit analysis they would undertake on a regular basis. Thus, two categories emerged within this major theme: *managing energy levels*, and *growing acceptance of exercise*.

### Managing energy levels

Issues related to battling fatigue and managing energy levels were consistently cited as the most challenging aspects of dealing with MS. There was a sense of ‘opportunity cost’, in that energy is a limited resource that had to be conserved. Participants felt that the energy and effort that is required to get through an exercise session has been spent and is therefore not available for other activities. Alex observed:

Exercise is always a challenge, to make the hurdle over the initial fatigue to get into it, get into a routine. On certain days, with the increased amount of fatigue associated with MS, I think it’s very difficult to get up and get motivated to get going and do it, and not to feel so tired that the rest of your day is lost because you put out a certain amount of energy to do a bit of exercise.

Similarly, Nora (spouse) noted:

It’s the energy, right? It’s the energy to be able to do it. People think, ‘if I have limited energy to do something, should I use that energy to go to the gym to work out or should I use that energy to pick my kid up after school and walk them to the park?’

The energy required simply to manage everyday activities is often significant, and thereby affects what else can be attempted in a day. Rob expressed these sentiments:

Fatigue is a fairly big symptom and I do get tired if I do too much at once. Early morning is not good for me because, frankly, getting dressed is a major accomplishment really. I sort of think ‘Ah, I’ve done it!’ but it takes so long because early morning things are a little bit difficult for me.

Due to the obstacles encountered, and to the issues related to energy management, engaging in exercise has often been challenging for people with MS. The historical ambivalence of some health professionals towards exercise due to concerns related to fatigue and heat sensitivity issues magnified the general reluctance expressed by people with MS to partake in physical activity [7, 8]. This way of thinking has changed considerably in recent years as emerging research has been more conclusive with respect to the benefits of exercise [13]. This change is reflected by our participants in the final category, *a growing acceptance of exercise.*

### A growing acceptance of exercise

Despite the recent evidence emphasizing the benefits of regular exercise, it has taken time to be fully embraced by those in the MS community. Old attitudes occasionally prevail, in the sense that exercise is still regarded as somewhat of a risky endeavour. Ben commented that it was his spouse (Louise) who took the initiative by signing him up for an exercise program, and he has now been exercising for more than 6 years. “If she hadn’t have signed us up for the program, I likely would not have gotten involved. Well, and also, it was counter-indicated by the MS clinic. I was told not to”. Rob mentioned the cautious nature of those advising him in the early stages.

I don’t think the neurologist was all for it at first for any of us, to tell you the truth. When you go there, you do see the physiotherapist and she gives you a lot of bending and stretching exercises. But the only strengthening exercise she ever gave me was sitting on a straight-back chair and getting up without using your hands. You were to do twenty of those a day or whatever. I did those for years and they did help a little but that was it. When I mentioned coming (to the exercise program) they didn’t really give me the impression they thought I should do it. I think that they felt it would cause too much fatigue and fatigue is a big, big problem.

On the other hand, Jeff acknowledged that the attitudes are changing, as more research studies emerge that support engaging in exercise as beneficial for people with MS:

I think it’s probably changed a little bit, there’s more literature out there, but I can say when I first started volunteering at the MS Society, exercise was a no-no and it was very hard to get any supporting evidence to do it.

Alex stated that attitudes were indeed starting to change, and he has witnessed a distinct shift in how exercise is being addressed. “That’s changing….this thing I went to recently, which was the 25^th^ anniversary at the clinic….exercise as part of your getting better was quite strongly stressed.”

## Discussion

Research has only recently started to explore the psycho-social impact of exercise for those with MS [14]. With the physical benefits now increasingly accepted and guidelines established [9], researchers have turned to examining both the multiple benefits and barriers as they pertain to various forms of physical activity for people with MS [14, 15, 31, 32]. Our results support and expand upon those findings, particularly with respect to inclusion of the perspectives of spouses. Specifically, all of the participants with MS found great physical benefits to exercise. One of our participants noted that he was growing increasingly reliant on a wheelchair prior to starting in his current exercise program, but he no longer requires one. Others described improvements that were slightly less dramatic, such as regaining the ability to do household chores, or visit with friends, but were nevertheless important advances to their physical functioning and quality of life.

The psychological benefits were also evident, and often emphasized by the spouses, with comments such as ‘he comes home happier’ and ‘he feels better about himself’. Intertwined with the psychological component was the social interaction, which constituted a third major benefit. This was clearly a meaningful aspect of exercise for our participants who were exercising in a group setting, all of whom indicated that they would find it exceedingly difficult to exercise on their own. This emerged as a notable variable in our findings; those involved in the group program emphasized the social component, while the participants who exercised on their own reported more difficulty in maintaining a regular exercise schedule. Borkoles and colleagues described similar sentiments in their interviewees, who remarked that the social element made exercise more enjoyable, and lessened their feelings of isolation [14]. We remain cautious about our findings, however, given that the three oldest participants were partaking in the group exercise program and thus the potential exists for an associated age confound. Further exploration of group-based exercise, and any additional benefits derived from such programs over exercising on one’s own, is a potentially valuable avenue for future research.

The barriers to exercise noted by our participants supported and reinforced those expressed in recent studies. Managing energy and minimizing fatigue has been consistently mentioned as a barrier to exercise for those with MS [14, 15, 32,] and conflicting beliefs about the value and benefits of exercise caused tension and uncertainty about involvement [32]. Certainly the concern that energy was limited, and thus a valuable resource important to conserve, emerged in our interviews. There was a distinct sense of opportunity cost that participants expressed due to the fear of not having enough energy post-workout to function in any sort of meaningful way in other aspects of their lives. The choices expressed by participants and their spouses were sometimes as stark as going to the gym to work out or using that energy to ‘pick my kid up after school and walk them to the park?’ Emerging evidence, however, emphasizes the positive benefits that can be derived from regular involvement in physical activity. Research advocating exercise for people with MS started to accumulate the 1990s [8] and that momentum has continued to build over the subsequent two decades. Recent reviews of the literature (e.g., [10, 13]) have clearly articulated the benefits of exercise to improve physical function and manage symptoms most closely associated with the disease. The evidenced-based guidelines developed by Latimer and colleagues [9] provide specific exercise recommendations for individuals with MS, and may help to alleviate concerns related to physical activity involvement.

Our research did not focus on sedentary individuals, nor individuals who had been exercising previously and then stopped. Future studies that gain insight into the opportunities for people with MS to partake in exercise, and the process by which individuals initiate and maintain involvement in physical activity would prove valuable. Importantly, issues related to accessibility remain a significant barrier to engagement in exercise. Transportation to an exercise venue and parking once there are potential impediments to physical activity, made worse by inclement weather. One of our participants mentioned the use of an ‘access bus’, which needed to be booked two weeks in advance, but due to irregular pick up and drop off times, ‘the fitness thing could end up taking five hours’. Learmonth and colleagues noted that their participants cited transportation as an important issue, whether they were relying on their own, or public transport [15]. For those living in rural communities, this becomes increasingly problematic. Home-based exercise provides one solution to transportation issues, but at the cost of the social component [14] and potentially an aspect of supervision, professional or otherwise, during workouts.

Our findings were enriched by the inclusion of interviews with the spouses, done separately from their partner with MS, and a virtually unexamined component of the exercise/MS experience. These interviews provided a fuller, more holistic picture of living with MS, and the extent to which exercise can be beneficial to the lives of both partners. What became clear was the potential for the spouse to play a facilitating role. This could come in multiple forms, such as support and encouragement to partake in physical activity, conquering accessibility barriers by aiding with transportation, or actually attending a gym or exercise class with their partner. One partner noted that she was ‘not sure that (Ben) would get up at 6:30 on his own twice a week if I weren’t going with him’.

The payoff to such facilitation appears to be significant. One of the major issues to emerge in previous research is the notion of independence, and concerns with maintaining that to the greatest extent possible in the face of the disease [13]. Exercise had a profound effect on the maintenance of independence with our study participants. The improving physical function allowed greater mobility around the home, and greater ability to contribute to various household tasks. It also allowed for greater independence of the spouse, by reducing the care-giving responsibilities, and by allowing them to go out without worrying whether their partner could fend for themselves alone in the home. Not surprisingly, MS can cause strain in a relationship, to the point where “supporting MS couples as they adapt to role changes and helping couples avoid relational strain is a stated MS research agenda item” [16]. Our findings suggest that exercise may play an integral part in maintaining independence and reducing strain on the relationship.

## Conclusion

Rather than an inexorable downward decline in physical ability that is common with MS, our participants spoke of a positive reversal in physical function, which has had far-reaching implications for multiple aspects of their lives, including their psychological outlook, their sense of independence, overcoming isolation, and their relationship with their spouse, all of which are identified in the literature as notable aspects of life affected by the disease. Spouses can often help to counteract barriers and facilitate exercise, and our interviews suggest they are well aware of the important role they play. Spouses also valued the increased independence they experienced, in the form of reduced care-giving responsibilities and enhanced social opportunities, as a result of the improved physical function of their partner. While MS brings strain to a relationship, this can be mitigated, to a certain extent, when exercise is regular and sustained. The expanding research on the psycho-social benefits of exercise for those with MS, as well as the recently published exercise guidelines will potentially aid in giving individuals both the tools and the confidence to engage in physical activity as a way of improving their quality of life.
